# Efficacy of Higher Positive End-Expiratory Pressure Ventilation Strategy in Patients With Acute Respiratory Distress Syndrome: A Systematic Review and Meta-Analysis

**DOI:** 10.7759/cureus.26957

**Published:** 2022-07-18

**Authors:** Ryohei Yamamoto, Sosuke Sugimura, Kazuki Kikuyama, Chihiro Takayama, Junichi Fujimoto, Koichi Yamashita, Yasuhiro Norisue, Chihiro Narita

**Affiliations:** 1 Department of Healthcare Epidemiology, School of Public Health, Graduate School of Medicine, Kyoto University, Kyoto, JPN; 2 Department of Medical Engineering, Faculty of Health Care Science, Himeji Dokkyo University, Hyogo, JPN; 3 Department of Intensive Care Medicine, Showa University Hospital, Tokyo, JPN; 4 Department of Intensive Care Medicine, Yokohama Rosai Hospital, Yokohama, JPN; 5 Division of Critical Care Center, Kochi Red Cross Hospital, Kochi, JPN; 6 Department of Emergency and Critical Care Medicine, Tokyo Bay Urayasu Ichikawa Medical Center, Urayasu, JPN; 7 Department of Emergency Medicine, Shizuoka General Hospital, Aoiku, JPN

**Keywords:** systematic review and meta analysis, invasive mechanical ventilation, peep, peep - positive end expiratory pressure, ventilator induced lung injury, severe ards, ards (acute respiratory distress syndrome), acute respiratory distress syndrome [ards]

## Abstract

Previous systematic reviews and meta-analyses assessing the pooled effects of higher positive end-expiratory pressure (PEEP) failed to show significantly reduced mortality in patients with acute respiratory distress syndrome (ARDS). Some new randomized controlled trials (RCTs) have been reported and an updated systematic review is needed to evaluate the use of higher PEEP in patients with ARDS.

We searched MEDLINE, Cochrane Central Register of Controlled Trials (CENTRAL), EMBASE, Cumulative Index to Nursing and Allied Health Literature (CINAHL), Igaku-Chuo-Zasshi, ICTRP, the National Institute of Health Clinical Trials Register, and the reference list of recent guidelines. We included RCTs to compare the higher PEEP ventilation strategy with the lower strategy in patients with ARDS. Two authors independently assessed the eligibility of the studies and extracted the data. The primary outcomes were 28-day mortality. The GRADE (Grading of Recommendations, Assessment, Development, and Evaluations) methodology was used to evaluate the certainty of the evidence.

Among the 6530 screened records, 16 randomized trials involving 4150 patients were included in our meta-analysis. When comparing higher PEEP versus lower PEEP, the pooled risk ratio (RR) for 28-day mortality was 0.85 (15 studies, n=4108, 95% CI 0.72 to 1.00, I^2^=58%, low certainty of evidence). Subgroup analysis by study participants with a low tidal volume (LTV) strategy showed an interaction (P for interaction, 0.001).

Our study showed that in patients with ARDS, the use of higher PEEP did not significantly reduce 28-day mortality compared to the use of lower PEEP.

## Introduction and background

Acute respiratory distress syndrome (ARDS) is a common clinical syndrome with substantial morbidity and mortality in the intensive care unit (ICU) [1]. Most patients with ARDS require mechanical ventilation and the mortality was 28-47.8% [2]. In the management of patients with ARDS, the application of positive end-expiratory pressure (PEEP) is one method to improve oxygenation in patients. PEEP prevents atelectasis, increases functional residual capacity, recruits alveoli, redistributes extravascular lung water, and improves ventilation-perfusion matching. The higher PEEP usually improves the PaO_2_/FiO_2_ ratio compared to a lower PEEP [3-7]. However, there is a potential risk of increasing dead space, carbon dioxide, and pressure injury.

The results of randomized controlled trials (RCTs) that evaluated the efficacy of higher PEEP were inconsistent [3-5,8-9]. The Cochrane Systematic Review of seven trials that involved 2,565 patients with ARDS showed that in-hospital mortality was not significantly reduced by higher PEEP [10]. Recent systematic reviews have demonstrated a trend toward a lower risk of mortality, but the difference was insignificant [6,11]. However, these studies did not include three RCTs published after 2018 [12-14].

Higher PEEP ventilation has potentially relevant benefits; however, the effects remain unclear. To update the Japanese ARDS guidelines 2016 [15], we conducted a systematic review and meta-analysis aimed to update and evaluate the efficacy of higher levels of PEEP in patients with ARDS.

## Review

Methods

Protocol and Registration

Our review followed the Preferred Reporting Items for Systematic Reviews and Meta-Analyses (PRISMA) protocol for RCTs. The protocol was submitted to the University Hospital Medical Information Network Clinical Trial Registry (UMIN-CTR) on July 12, 2020, before data extraction was initiated (identifier: UMIN000041074).

Eligibility Criteria

Randomized controlled trials (RCTs) or cluster RCTs were included. Crossover trials, quasi-randomized, and non-randomized trials were excluded. The target population included intubated patients with ARDS (age ≥16 years). ARDS was defined according to the Murray [16], American-European Consensus Conference (AECC) [17], and Berlin definition [18], or definitions determined by the authors in the study. We included studies that compared a higher PEEP ventilation strategy with the usual or lower PEEP ventilation strategy; a variety of PEEP settings were included. If there was a difference of more than 2 cmH_2_O in PEEP between the two groups during 24-72 hours after intervention due to differences in the methods of setting PEEP (FiO_2_/PEEP, targeted O_2_, research-defined protocols, programmatic algorithm, and PEEP titration by recruitment maneuver, compliance, and transpulmonary pressure), we included them in this review. Differences in the settings of the ventilation mode and target tidal volume were tolerated and incorporated. Because the effect of high-frequency oscillatory ventilation and airway pressure release ventilation were different from our scope, we excluded those studies that evaluated them.

Data Sources and Searches

We conducted a literature search of the Cochrane Central Register of Controlled Trials (CENTRAL), MEDLINE through PubMed, EMBASE, Cumulative Index to Nursing and Allied Health Literature (CINAHL), and Igaku-Chuo-Zasshi (Ichu-shi) from inception until July 2020. Our search strategies are described in the appendices. We also searched for ongoing clinical trials in the following trial registries: The World Health Organization International Clinical Trials Registry Platform (http://apps.who.int/trialsearch/) and the United States National Institutes of Health Ongoing Trials Register ClinicalTrials.gov. software (www.clinicaltrials.gov). We searched for reference lists of the guidelines on the management of ARDS and the extracted articles [6,15]. No language restrictions were applied to these searches.

Study Selection and Data Extraction

Titles and abstracts were independently evaluated for potential relevance by two reviewers (SS, JF, KY, KK, CT, and RY). Two reviewers independently assessed the full texts and study eligibility and verified the reasons for the exclusion of ineligible studies. Conflicts between two reviewers were resolved by discussion and where this failed, through arbitration by a third author (CN). Following the PRISMA statement, the selection process was recorded in a flow diagram with appropriate details. Data extraction was performed by two authors (SS, JF, KY, and RY) independently using standard data extraction sheets. Differences in opinions regarding data extraction were resolved using the same methods.

Type of Outcome Measures

The primary outcome was 28-day mortality. If a survival curve was available, we extracted the 28-day mortality. If 28-day mortality was not reported, we used the mortality of the nearest follow-up at 28 days. The secondary outcomes were longest follow-up mortality (longest follow-up regardless of the duration of follow-up employed), health-related quality of life (QOL), PaO_2_/FiO_2_ (P/F) ratio on day one, ventilation-free day (VFD) up to 28 days, hospital length of stay (LOS), and barotrauma (new pneumothorax, mediastinal emphysema, subcutaneous emphysema, new thoracic drainage, or author-defined barotrauma/ventilation-induced lung injuries (VILI)).

Assessment of Risk of Bias

Two reviewers independently assessed the risk of bias in the included studies using the Cochrane Collaboration tool for assessing the risk of bias in randomized trials [19]. These reviewers classified each potential risk of bias as “low,” “high,” or “unclear.” Disagreements between the reviewers regarding the risk of bias were resolved through discussion. If failed, a third reviewer (CN) resolved these disagreements.

Analysis and Results Synthesis

Measures of treatment effects were calculated using the Cochrane Review Manager software (version 5.3; Cochrane Collaboration, London, UK) for data synthesis and analysis. Dichotomous data (mortality, barotrauma) were expressed as RR with 95% CIs, and continuous data (QOL, VFD, LOS) were presented as mean difference (MD) with 95% CI.

The random-effects model was used for meta-analysis. The chi^2^ test and the I^2^ statistic were used to measure heterogeneity. We considered that a P-value of less than 0.1 and greater than 60% in the chi^2^ and I^2^ statistic tests, respectively, were significantly heterogeneous. To evaluate publication bias, funnel plots and Egger’s tests were performed, where each comparison included more than ten studies. Egger’s test was considered statistically significant at P< 0.05.

Subgroup Analysis and Sensitivity Analysis

We pre-planned the following subgroup analyses for primary outcomes (28-day mortality): the Berlin definition or not, LTV strategy (LTV in the comparison group or not), and P/F ratio ≤200 mmHg at the time of inclusion. We performed sensitivity analyses that included only studies with a low risk of bias to evaluate the robustness of our inferences for the primary outcomes.

Assessment of the Certainty of the Evidence

To evaluate the quality of the evidence, the Grading of Recommendations Assessment, Development, and Evaluation (GRADE) considerations were used classifying the quality as “high,” “moderate,” “low,” or “very low.” We used the methods and recommendations described in the Cochrane Handbook for Systematic Reviews of Intervention [20].

Results

Study Selection

**Figure 1:**
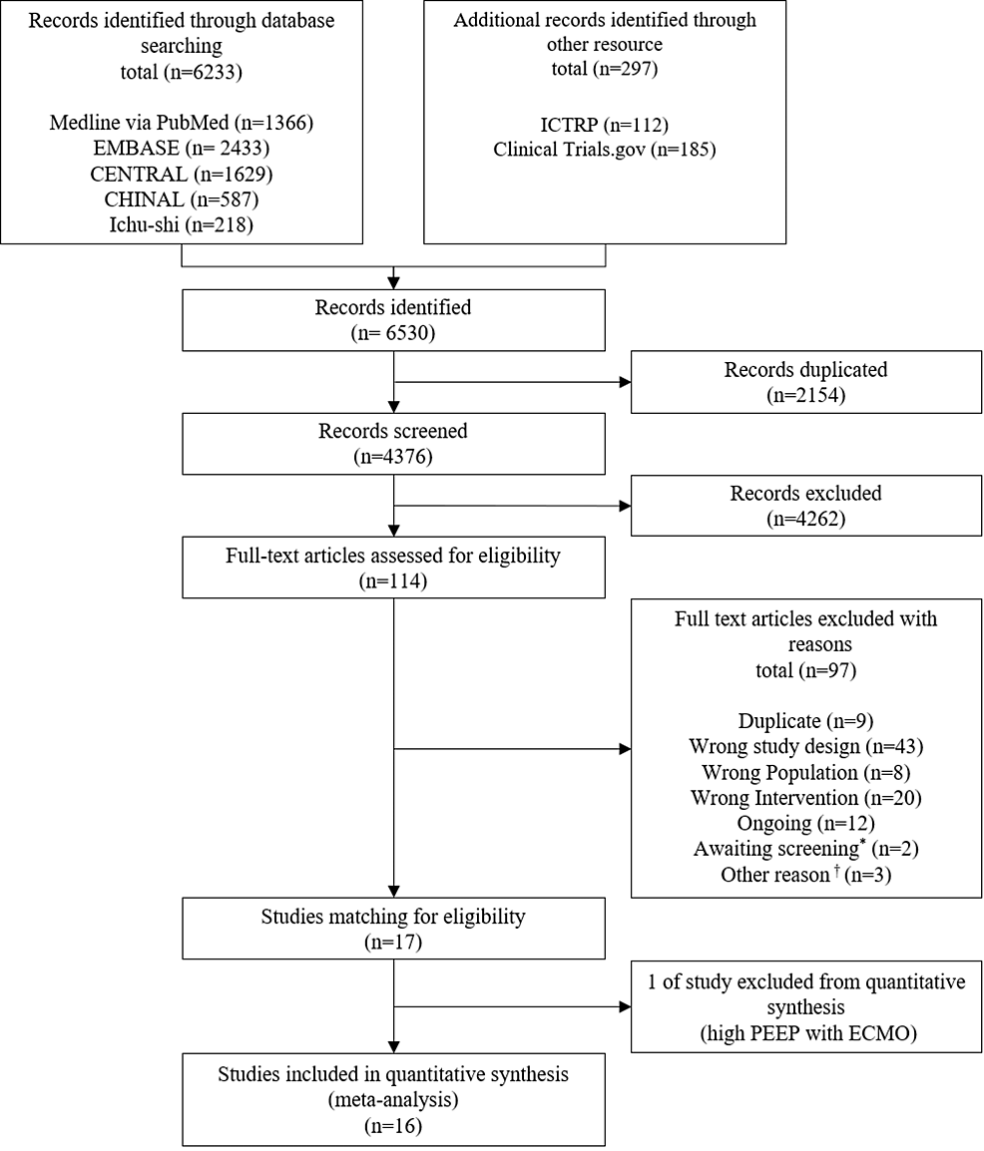
PRISMA flow diagram ^*^ One record did not describe post-intervention PEEP levels, and one record was of unclear design. ^†^ Two records were interim analyses reports, one record was the first report of Amato 1998 [8]. PRISMA: Preferred Reporting Items for Systematic Reviews and Meta-Analyses; CENTRAL: Cochrane Central Register of Controlled Trials; CINAHL: Cumulative Index to Nursing and Allied Health Literature; ICTRP: International Clinical Trials Registry Platform; PEEP: positive end-expiratory pressure; ECMO: extracorporeal membrane oxygenation

After screening 6530 references, 114 records were evaluated in detail and 97 were excluded (Figure 1). Seventeen RCTs met the inclusion criteria [3-5,8-9,12-14,21-28], and one trial assessed the effects of higher PEEP during extracorporeal membrane oxygenation (ECMO) [29]. Therefore, 16 randomized trials involving 4150 patients were included in the quantitative synthesis to compare higher PEEP versus lower PEEP.

Study Characteristics

The characteristics of this study are summarized in Table 1. ARDS was defined by the lung injury severity score (LISS) [8], AECC criteria [3,5,9,21-26], Berlin definition [12-14,27-29], and the author's definition [4]. Most studies used the P/F ratio as an inclusion criterion. The intervention procedure varied across the studies. PEEP adjustments in the intervention groups were performed using the PEEP table in three of 16 studies [3-4,22], PEEP above the lower inflection point of the pressure-volume curve of the respiratory system (Pflex) in three of 16 studies [8-9,21], and compliance, including the decremental PEEP method, in four of 16 studies [13,24-26]. The mean level of PEEP on the first day in the high PEEP group ranged from 10.0 to 18.7 cm H_2_O, and in the control group, it was 6.8-12.0 cm H_2_O. The levels of PEEP on Days 1, 3, and 7 are described in Table 2.

**Table 1:** Level of PEEP on Days 1, 3, and 7 * 36 hours; † Two to three hours PEEP: positive end-expiratory pressure; SE: standard error; SD: standard deviation; CI: confidence interval; IQR: interquartile range; NA: no data

Study	Level of PEEP (cm H_2_O)						
		Day 1		Day 3		Day 7	
	Scale	Inttervention	Control	Intervention	Control	Intervention	Control
Amato 1998 [8]	mean (SE)	16.3 (0.7) *	6.9 (0.8) *	NA	NA	13.2 (0.4)	9.3 (0.5)
Ranieri 1999 [21]	mean (SD)	14.8 (2.7) †	6.5 (1.7) †	NA	NA	NA	NA
Brower 2004 [3]	mean (SD)	14.7 (3.5)	8.9 (3.5)	12.9 (4.5)	8.5 (3.7)	12.9 (4.0)	8.4 (4.3)
Long 2006 [22]	NA	NA	NA	16 (3)	11 (2)	15 (6)	11 (3)
Villar 2006 [9]	mean (SD)	14.1 (2.8)	9.0 (2.7)	11.2 (3.1)	8.7 (2.8)	NA	NA
Meade 2008 [4]	mean (SD)	15.6 (3.9)	10.1 (3.0	11.8 (4.1)	8.8 (3.0)	10.3 (4.3)	8.0 (3.1)
Mercat 2008 [5]	mean (SD)	14.6 (3.2)	7.1 (1.8)	13.4 (4.7)	6.7 (1.8)	8.9 (5.1)	6.2 (2.1)
Talmor 2008 [23]	mean (SD)	18.7 (5.1)	11	17 (6)	10 (4)	NA	NA
Hodgson 2011 [24]	mean (SD)	15 (1)	10 (0.5)	12.1 (1.5)	9.3 (1.4)	8.5 (1.8)	7.8 (2.0)
Kacmareg 2016 [25]	mean (SD)	15.8 (3.8)	11.6 (2.5)	14.3 (3.9)	10.7 (3.3)	11.2 (4.4)	10.5 (3.9)
Li 2017 [27]	mean (SD)	19.2 (NA)	6.8 (2.1)	NA	NA	NA	NA
ART 2017 [26]	mean (95%CI)	16.2 (15.9 to 16.6)	12.0 (11.7 to 12.3)	14.2 (13.8 to 14.6)	10.5 (10.2 to 10.9)	11.6 (11.2 to 12.1)	9.6 (9.3 to 10.0)
Nguyen 2019 [13]	mean (SE)	14.8 (0.3)	10.3 (0.5)	9.9 (0.5)	10.9 (0.5)	8.5 (0.5)	10 (0.8)
Hodgson 2019 [24]	mean (SD)	16.1 (3.6)	11.3 (4)	13.3 (4.9)	10.8 (4.9)	9.8 (3.6)	10.3 (4.6)
Wang. B 2019 [28]	mean (SD)	10 (4.5)	8 (5)	12 (2.9)	6.5 (4.5)	NA	NA
Salem 2020 [14]	median [IQR]	10 [8–13.5]	8 [8–10]	NA	NA	NA	NA
Wang. R 2020 [29]	mean (SD)	15.7 (3.4)	12.5 (2.8)	14.6 (3.2)	11.8 (2.6)	NA	NA

**Table 2:** Characteristics of selection studies ARDS: acute respiratory distress syndrome; AECC: American-European Consensus Conference; ICU: intensive care unit; P/F: PaO_2_/FiO_2_; PEEP: positive end-expiratory pressure; LISS: lung injury severity score; Pflex: lower inflection point of pressure-volume curve; Pplat: plateau pressure during an inspiratory pause; Ppeak: peak pressure; Ptp: transpulmonary pressure; Vt: tidal volume; BW: body weight; PBW: predicted body weight; IBW: ideal body weight; VCV: volume-controlled ventilation; PCV: pressure-controlled ventilation; NA: no data * Severity used in inclusion criteria

Study	No. of center	Definition of ARDS	*Severity P/F ratio	Intervention	Control	Mortality Outcome
Higher PEEP vs Lower PEEP						
Amato 1998 [8]	2	LISS	NA	PEEP at Pflex plus 2 cm H_2_O, Vt 6 ml/kg BW, PCV	PEEP set to O_2_ goals (FI0_2_ < 60 %), Vt 12ml/kg BW, VCV	ICU, 28-day, In-hospital
Ranieri 1999 [21]	2	AECC	≤200	PEEP at Pflex plus 2 to 3 cm H_2_O, Vt 5-8 mL/kg IBW, VCV	PEEP titrated by PEEP trial, Pplat<35cmH_2_O, VCV	28-day
Brower 2004 [3]	23	AECC	≤300	PEEP table (Higher PEEP group), Vt 6ml/kg PBW, VCV	PEEP table (Lower-PEEP group), Vt 6ml/kg PBW, VCV	30-day (from figure), In-hospital
Long 2006 [22]	1	AECC	NA	PEEP table, Vt 6ml/kg PBW, VCV	PEEP titrated static P-V curve, Vt<8 ml/kg IBW, VCV	28-day
Villar 2006 [9]	8	AECC	≤200	PEEP at Pflex plus 2 cm H_2_O, Vt of 5-8 mL/kg PBW, PCV	PEEP≥5 cm H_2_O, Vt 9-11 mL/kg PBW, VCV	ICU, 30-day (from figure), In-hospital
Meade 2008 [4]	30	Author's definition	≤250	PEEP table, Vt 4-8ml/kg PBW, PCV	PEEP table (lower), Vt 4-8ml/kg PBW, VCV	ICU, 28-day, In-hospital
Mercat 2008 [5]	37	AECC	≤300	PEEP titrated by airway pressure (<Pplat 28 to 30 cm H_2_O), Vt 6ml/kg PBW, VCV	PEEP 5-9 cm H_2_O, Vt 6ml/kg PBW, VCV	28-day, In-hospital
Talmor 2008 [23]	1	AECC	≤300	PEEP titrated by Ptp (0 to 10 cm H_2_O), Vt 6±2 ml/kg PBW, VCV or PCV	PEEP table (ARDS net, lower), Vt 6±2 ml/kg PBW, VCV or PCV	28-day
Hodgson 2011 [24]	1	AECC	<300	PEEP at decremental PEEP plus 2.5cm H_2_O, Vt≤6ml/kg PBW, PCV	PEEP table (ARDS net, lower), Vt≤6ml/kg PBW, VCV	in-hospital
Kacmareg 2016 [25]	20	AECC	≤200	PEEP at decremental PEEP plus 3cm H_2_O, Vt 6ml/kg PBW, PCV	PEEP table, Vt 6ml/kg PBW, VCV	ICU, 28-day, In-hospital
Li 2017 [27]	2	Berlin definition	≤200	PEEP set according to Ptp 10cmH_2_O, Vt 6ml/kg,	PEEP table, Vt 6ml/kg	28-day
ART 2017 [26]	120	AECC	≤200	PEEP at maximum alveolar recruitment by compliance plus 2 cmH_2_O, Vt 4-6ml/kg PBW, Pplat ≤30cmH_2_O, VCV	PEEP table (ARDS net, lower), Vt 4-6ml/kg PBW, Plateau ≤30cmH_2_O, VCV	ICU, 28-day, In-hospital
Nguyen 2019 [13]	1	Berlin definition	≤200	PEEP at the best compliance plus 2cmH_2_O, Vt 6ml/kg, VCV	PEEP table (ARDS net, lower), Vt 6ml/kg, VCV	28-day
Hodgson 2019 [24]	35	Berlin definition	≤200	PEEP at the level of desaturation plus 2.5 cm H_2_O, Vt 4-6ml/kg, PCV	PEEP table (ARDS net, lower), Vt 6ml/kg, VCV	ICU, 28-day, 90-day, In-hospital
Wang. B 2019 [28]	1	Berlin definition	NA	PEEP titrated by esophageal pressure (5 to 8 cm H_2_O), Vt 6-8 mL/kg IBW, VCV	PEEP table (ARDS net, lower), Vt 6ml/kg IBW, VCV	NA
Salem 2020 [14]	1	Berlin definition	NA	PEEP titrated by LUS aeration score, Vt 4-8ml/kg PBW, VCV	PEEP table (ARDS net, lower), Vt 4-8ml/kg PBW, VCV	28-day
Higher PEEP vs Lower PEEP with ECMO						
Wang. R 2020 [29]	1	Berlin definition	≤80	PEEP titrated by expiratory Ptp (0 and 5 cm H_2_O), Ppeak less than 25 cm H_2_O, PCV	PEEP 10-15 cm H_2_O, Ppeak less than 20-25 cm H_2_O, PCV	30-day (from figure), 60-day

Risk of Bias Assessment

**Figure 2:**
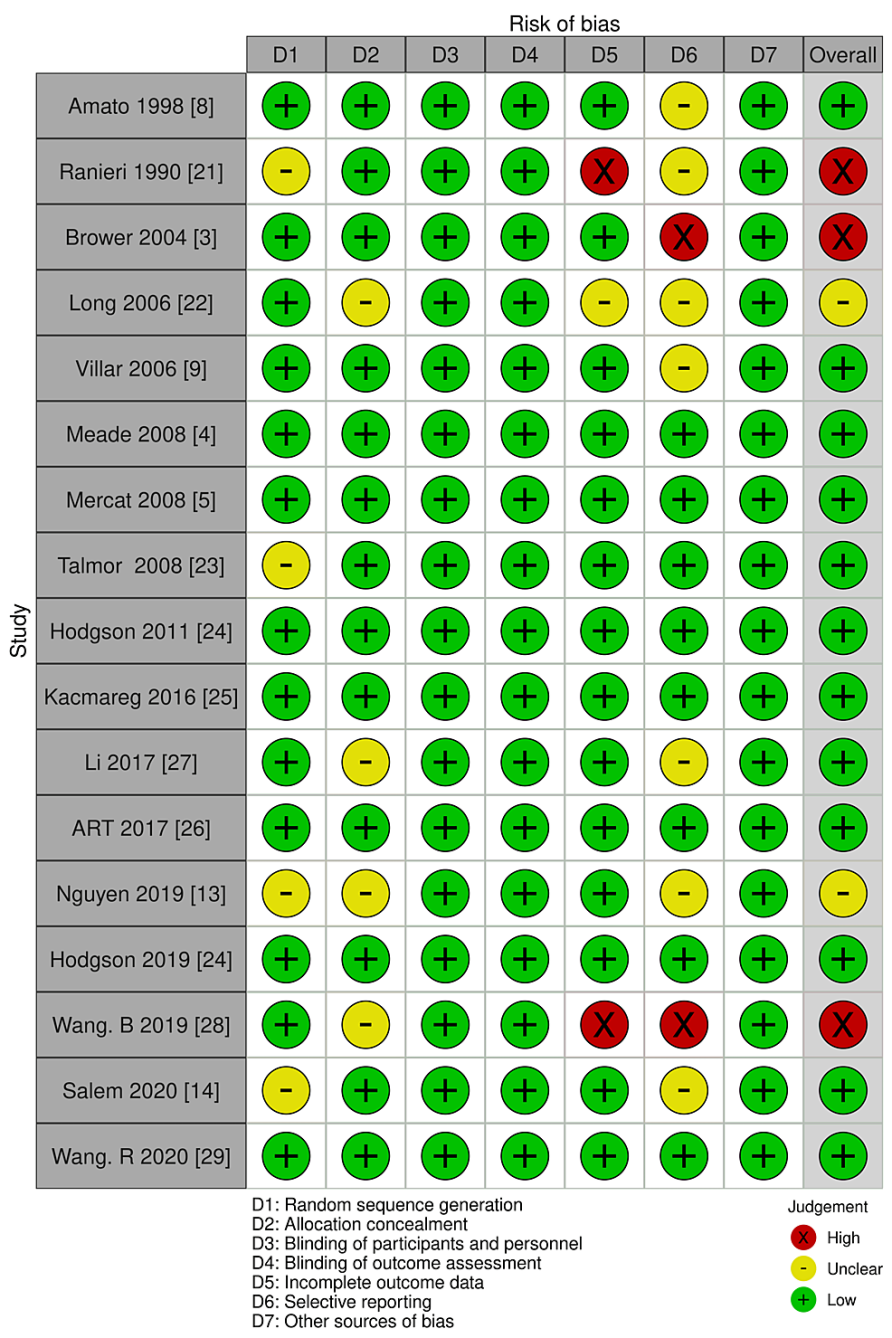
Risk of bias summary

**Figure 3:**
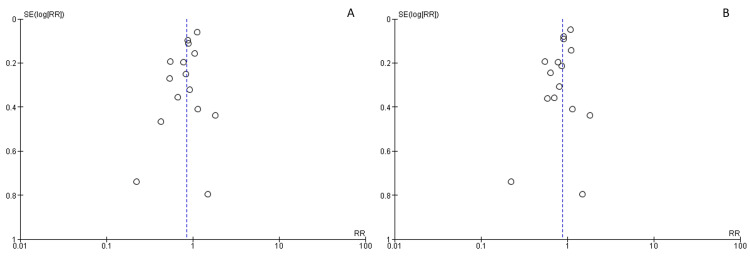
Funnel plot for mortality SE (log [RR]): standard error (log [risk ratio]); RR: risk ratio A: 28-day mortality; B: Longest follow-up mortality

The risk of bias for mortality was “low” when comparing higher PEEP and lower PEEP (Figure 2). Blinding was not performed because of the nature of the intervention in all studies. Because unblinding does not influence objective outcomes, we evaluated mortality and P/F ratio as a low risk of bias. While subjective outcomes, such as QOL, VFD up to 28 days, hospital LOS, and barotrauma were assessed as “unclear” risk of bias related to blinding. For incomplete outcome data, one study was evaluated as “high” risk of bias due to the exclusion of seven patients after randomization [21]. For selective outcome reporting, studies for which protocols were not available or details could not be obtained at the trial registration site were rated as "unclear" risk of bias. In Brower, the protocol was changed during the trial, and the primary endpoint was switched from "28-day mortality" to "proportion of patients who died before discharge without respiratory support" [3]. Therefore, we evaluated the risk of bias as “high;” no publication bias was detected (Figure 3).

Meta-Analyses of the Results

**Figure 4:**
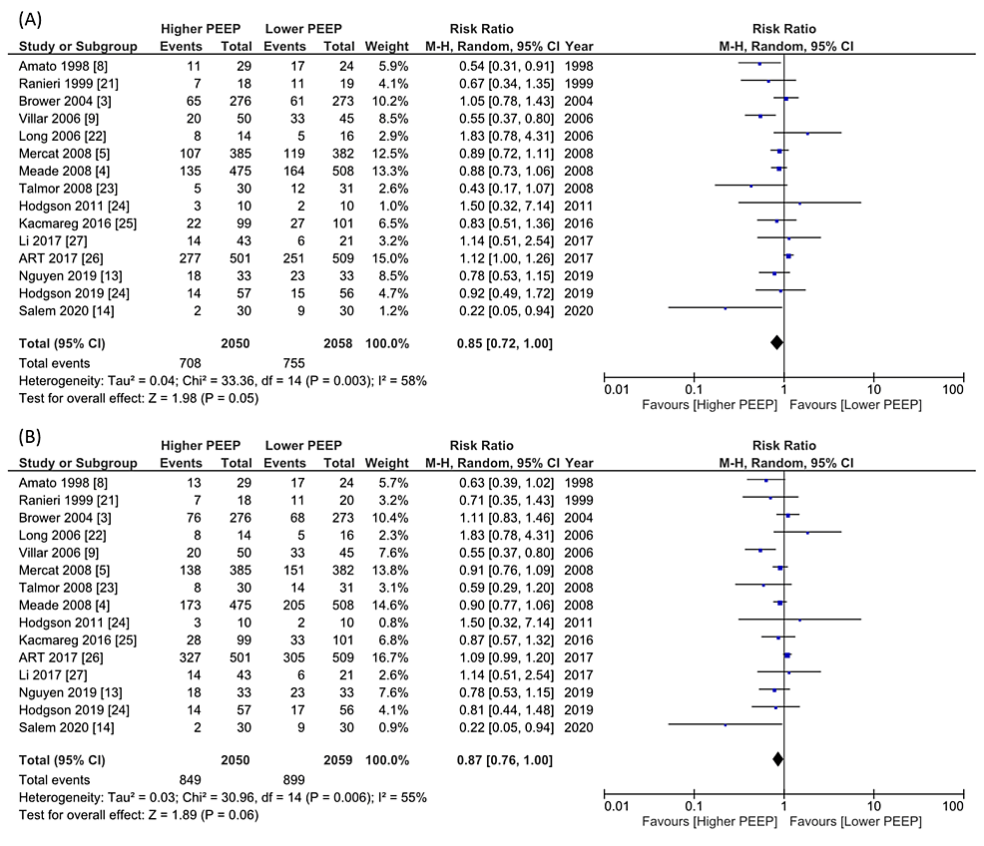
Forest plot of the comparison of higher PEEP ventilation versus lower PEEP ventilation for mortality PEEP: positive end-expiratory pressure; CI: confidence interval; M–H: Mantel-Haenszel method (A) 28-day mortality. Data extracted from the Kaplan-Meier curve at 28 days; Brower 2004 [3], and Villar 2006 [9], in-hospital mortality; Hodgson 2011 [24], and 28-day mortality; the other studies. (B) The longest follow-up mortality. Data extracted from the Kaplan-Meier curve at 28 days; Villar 2006 [9], 28-day mortality; Ranieri 1999 [21], Long 2006 [22], Li 2017 [27], Nguyen 2019 [13], and Salem 2020 [14], 60-day mortality; Brower 2004 [3], Mercat 2008 [5], and Kacmareg 2016 [25], six-month mortality; Talmor 2008 [23], ART 2017 [26], and Hodgson 2019 [24], and in-hospital mortality; other studies.

**Figure 5:**
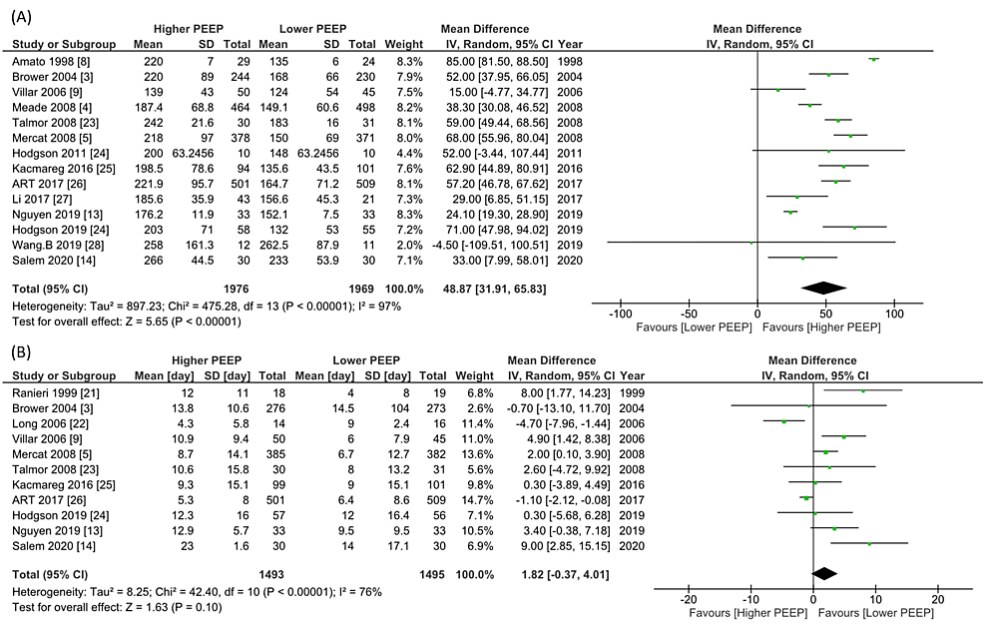
Forest plot of the comparison of higher PEEP ventilation versus lower PEEP ventilation for P/F ratio at day1 and VFD up to 28 days. CI, confidence interval; IV, inverse variance; PEEP: positive end-expiratory pressure. (A): PaO_2_/FiO_2_ ratio on day 1; (B) Ventilator-free days up to 28 days.

**Figure 6:**
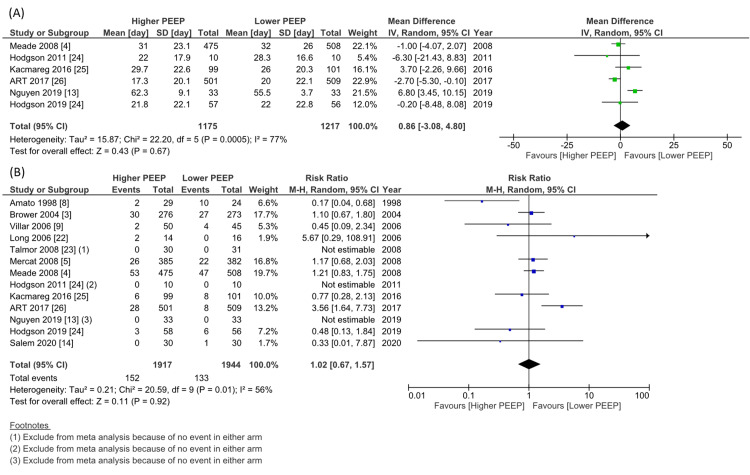
Forest plot of the comparison of higher PEEP ventilation versus lower PEEP ventilation for the length of hospital stay and barotrauma PEEP: positive end-expiratory pressure; CI: confidence interval; IV: inverse variance; M–H: Mantel–Haenszel method. (A) Length of hospital stay; (B) Barotrauma.

Regarding the primary outcome (28-day mortality), the pooled RR was 0.85 (15 studies, n=4108, 95% CI 0.72 to 1.00, I^2^=58%, Figure 4A). For the 4108 patients involved in the 15 studies and contributing to the longest follow-up mortality data, the pooled RR was 0.87 (15 studies, 95% CI 0.76 to 1.00, I^2^=55%; Figure 4B). None of the studies reported QOLs. Fourteen studies involved 3945 patients that reported a P/F ratio on day one; higher PEEP significantly increased the P/F ratio compared to lower PEEP (MD 48.9 mmHg, 95% CI 31.9 to 65.8, I^2^=97%, Figure 5A). There was no significant difference in VFD up to 28 days (11 studies, n=2988, MD 1.82 days, 95% CI -0.37 to 4.01, I^2^=76%, Figure 5B), hospital length of stay (6 studies, n=2392, MD 0.86 days, 95% CI -3.08 to 4.80, I^2^=77%, Figure 6A), or barotrauma (13 studies, n=3861 RR 1.02, 95% CI 0.67 to 1.57, I^2^=56%, Figure 6B).

Subgroup and Sensitivity Analyses

Subgroup analyses of 15 studies reporting 28-day mortality [3-5,8-9,12-14,21-27] by study participants with a definition of ARDS (Berlin vs. other) and 12 studies reporting 28-day mortality [3-5,9,12,13,21,23-27] by study participants with the severity of inclusion criteria (P/F ratio≤200 vs. >200) did not show any subgroup interaction (Table 3). The subgroup analysis of 15 studies reporting 28-day mortality [3-5,8-9,12-13,21-27] by study participants with LTV strategy (LTV in the comparison group or not) demonstrated a significant interaction in mortality (test for subgroup differences: Chi^2^ = 10.33, P for interaction 0.001; Table 3).

**Table 3:** Subgroup analyses of 28-day mortality related to higher PEEP ventilation PEEP: positive end-expiratory pressure; P/F: PaO_2_/FiO_2_ ratio; LTV: low tidal volume.

Subgroup	Studies	Patients	Pooled risk ratio	I^2^ (%)	p-value
Participants: definition	15	4108		0	0.77
Berlin definition	4	303	0.81 [0.55, 1.18]		
Other definition	11	3805	0.86 [0.72, 1.03]		
Participants: severity	12	3965		0	0.38
P/F ratio≤200	8	1646	0.80 [0.61, 1.05]		
P/F ratio>200	4	2319	0.92 [0.81, 1.04]		
Participants: LTV		4108		90.3	0.001
LTV in both group	12	3923	0.94 [0.82, 1.09]		
Without LTV in comparison	3	185	0.56 [0.42, 0.75]		

We performed a sensitivity analysis exploring the influence of a high risk of bias. Two of the 15 studies contributing to 28-day mortality were rated as having a high risk of bias. The pooled RR was similar to that of the primary analysis (13 studies, n=3522, RR 0.83, 95%CI 0.69 to 1.00, I^2^=63%). In a post-hoc meta-analysis, limited to studies conducted after 2000, we found that the pooled RR was 0.89 (13 studies, n=4018, 95%CI 0.75 to 1.04, I^2^=57%).

Certainty of Evidence

The certainty of evidence for mortality was downgraded by two levels for inconsistency (different direction of effect in studies and high heterogeneity) and imprecision (wide confidence interval) and was considered low. The details of the other outcomes are presented in Table 4.

**Table 4:** Summary of findings PEEP: positive end-expiratory pressure; RCT: randomized control trial; ADL/QOL: activities of daily living/quality of life; P/F: PaO_2_/FiO_2_ ratio; CI: confidence interval; RR: risk ratio; MD: mean difference. a. different direction of effect in studies (null and effective); b. high I^2^ statistics and heterogeneity test was significant; c. Wide Confidence Interval; d. Different directions of effect in studies (effective, null, adverse); e. Not reached optimum information size (OIS).

Certainty assessment							No of patients		Effect		Certainty
No of studies	Study design	Risk of bias	Inconsistency	Indirectness	Imprecision	Other considerations	Higher PEEP	Lower PEEP	Relative (95% CI)	Absolute (95% CI)	
Mortality (nearest 28 days)											
15	RCT	not serious	serious ^a, b^	not serious	serious ^c^	none	708/2050 (34.5%)	755/2058 (36.7%)	RR 0.85 (0.72 to 1.00)	55 fewer per 1,000 (from 103 fewer to 0 fewer)	⨁⨁◯◯ LOW
Mortality (Longest follow up)											
15	RCT	not serious	serious ^a, b^	not serious	serious ^c^	none	849/2050 (41.4%)	899/2058 (43.7%)	RR 0.87 (0.76 to 1.00)	52 fewer per 1,000 (from 105 fewer to 0 fewer)	⨁⨁◯◯ LOW
ADL/QOL - not reported											
-	-	-	-	-	-	-	-	-	-	-	-
P/F ratio at day1											
14	RCT	not serious	serious ^a, b^	not serious	not serious	none	1976	1969	-	MD 48.87 mmHg higher (31.91 higher to 65.83 higher)	⨁⨁⨁◯ MODERATE
Ventilator free days at 28											
11	RCT	not serious	very serious ^b, d^	not serious	serious ^c^	none	1493	1495	-	MD 1.82 day higher (0.37 lower to 4.01 higher)	⨁◯◯◯ VERY LOW
Length of hospital stay											
6	RCT	not serious	very serious ^b, d^	not serious	serious ^c^	none	1175	1217	-	MD 0.86 days higher (3.08 lower to 4.8 higher)	⨁◯◯◯ VERY LOW
Barotrauma											
10	RCT	not serious	very serious ^b, d^	not serious	very serious ^c, e^	none	152/1917 (7.9%)	133/1944 (6.8%)	RR 1.02 (0.67 to 1.57)	1 more per 1,000 (from 23 fewer to 39 more)	⨁◯◯◯ VERY LOW

Discussion

Summary of Key Findings

The current systematic review and meta-analysis showed that a higher PEEP ventilation strategy did not significantly reduce the 28-day and longest follow-up mortality of adult patients with ARDS. While the P/F ratio was increased in the higher PEEP group, there was no significant effect on VFD at 28 days and hospital LOS. The higher PEEP ventilation strategy did not appear to significantly increase the risk of barotrauma.

Relation With Previous Systematic Reviews

Previous systematic reviews of the effect of higher PEEP on patients with ARDS have not reported a significant mortality benefit with high PEEP [11,26]. Our systematic review included several recent RCTs [12-14,25-27], but the results remained unchanged. A sensitivity analysis that excluded studies with a high risk of bias showed similar results. However, these results should be carefully interpreted. This was because of the high heterogeneity in the included studies. In some RCTs, the control group had non-LTV ventilation, and the intervention group had open-lung strategy ventilation that combined higher PEEP with LTV. This heterogeneity may be important for interpreting the results. In our review, we conducted a subgroup analysis to examine whether there was an interaction between LTV and higher PEEP. This subgroup analysis suggested that there was an interaction when combined with LTV. Walkey et al. reported similar results to our analysis, which involved a recent RCT [6]. These results suggest that not only high PEEP but also a combination of LTV may be more effective. However, since the main effect was not significant in this study, the results of the subgroup analysis must be interpreted with caution.

We performed a subgroup analysis of the definition of ARDS and differences in severity at inclusion (P/F≤200) and examined the interaction on the effect of higher PEEP, but found no interaction on mortality. A meta-analysis of individual patient data from three trials [3-5] by Briel et al. showed that patients with ARDS (P/F≤200 mmHg) may benefit from higher PEEP than patients with mild ARDS [7]. There are several possible reasons for this difference in results; one reason is that we performed a subgroup analysis by P/F ratio at the inclusion criteria. To maintain the randomness of covariates between subgroups, it is necessary to perform subgroup analysis with or without the inclusion criteria of severity or stratified randomization by severity. Briel et al. adjusted for confounders to equalize the groups, but this may have been insufficient [7]. The second reason is the difference in the number of included studies. In our study, we included 12 RCTs in the subgroup analysis. Under the assumption that there was no bias, the pooled effect size would have been closer to the true value if the number of included studies increased. However, the sample size was insufficient for detecting small effects in the subgroups in our study. Until further evidence is accumulated, it is not clear whether higher PEEP should be applied depending on the severity of the target patients.

Implications for Practice and Further Study

Higher PEEP did not show a significant reduction in mortality, but it did show a consistent effect on improving oxygenation [10-11,26]. A higher PEEP usually improves the PaO_2_/FiO_2_ compared to a lower PEEP. PEEP prevents atelectasis, increases functional residual capacity, and improves ventilation-perfusion matching. Our study also showed that oxygenation could be improved without increasing pressure injury. In the intervention groups of most of the included studies, the combination of higher PEEP with LTV did not increase the occurrence of clinically objectified barotrauma. Therefore, a high PEEP may be safer for patients with ARDS when combined with LTV.

It is not clear from this study which method of setting PEEP was the best; there were many different methods of setting PEEP in each study, including oxygenation, stress index, transpulmonary pressure, decremental PEEP adjustment after a recruitment maneuver, or pressure-volume curves. These subgroup analyses could not be performed because of the small number of studies.

Limitations

The most important limitation was heterogeneity. There was variation in PEEP levels in the intervention group (PEEP at Day 1; 10-16.3 cmH_2_O). In the control group, there was a similar variation in PEEP levels (PEEP at Day 1; 6.5-12.0 cmH_2_O). The setting of the target PEEP level and the PEEP level of the control population may affect the effect of higher PEEP. Second, the P/F ratios of patients at inclusion also varied. Therefore, we performed a subgroup analysis using P/F ratios and found no interaction. Third, the definition of ARDS also varied among the studies, and subgroup analysis by ARDS definition did not show any interaction. Fourth, there may have been variabilities in important common interventions such as recruitment maneuvers and prone positions. The key common interventions were not specified by the studies; we could not verify whether there was an interaction between these subgroups. A meta-analysis by Walkey et al. reported that they did not detect any interaction by performing a protocolized recruitment maneuver [6].

## Conclusions

Low‐certainty evidence showed that the use of higher PEEP compared to the use of lower PEEP did not reduce mortality. However, the use of higher PEEP in patients with ARDS might be justified because it improves oxygenation without increasing harm and is associated with reduced mortality when combined with LTV. Further studies are needed to determine which subpopulations with higher PEEP are effective.

## Appendices

MEDLINE via PubMed search strategy (until July 8, 2020）

#1 Respiratory Distress Syndrome, Adult[mh] OR ARDS[tiab] OR shock lung[tiab]

#2 acute respiratory distress[tiab] OR acute respiratory failure[tiab]

#3 Acute[tiab] AND ((respirat*[tiab] OR ventilat*[tiab] OR pulmon*[tiab]) AND (fail*[tiab] OR depression[tiab]))

#4 Lung injury[mh] OR ALI[tiab] OR Acute lung injur*[tiab] OR Ventilator-Induced Lung Injury[tiab]

#5 Respiratory insufficiency[mh] OR Respiratory insufficiency[tiab]

#6 Acute chest syndrome[mh] OR Acute chest syndrome[tiab]

#7 #1 OR #2 OR #3 OR #4 OR #5 OR #6

#8 Positive-Pressure Respiration[mh] OR Positive Pressure Respiration[tiab] OR Positive End-Expiratory Pressure[tiab] OR PEEP[tiab]

#9 Continuous Positive Airway Pressure[tiab] OR CPAP[tiab] OR NCPAP[tiab]

#10 Airway Pressure Release Ventilation[tiab] OR APRV[tiab]

#11 Intermittent Positive-Pressure Breathing[tiab] OR IPPB[tiab]

#12 Intermittent Positive-Pressure Ventilation[tiab] OR IPPV[tiab]

#13 lung protective ventilatory strateg*[tiab] OR LPVS[tiab]

#14 alveolar recruit*[tiab] OR recruitment maneuve*[tiab]

#15 #8 OR #9 OR #10 OR #11 OR #12 OR #13 OR #14

#16 #7 AND #15

#17 (randomized controlled trial[pt] OR controlled clinical trial[pt] OR randomized[tiab] OR placebo[tiab] OR clinical trials as topic[mesh:noexp] OR randomly[tiab] OR trial[ti]) NOT (animals[mh] NOT humans [mh])

#18 #16 AND #17

CENTRAL search strategy (until July 8, 2020）

#1 [mh "Respiratory Distress Syndrome, Adult"] OR ARDS:ti,ab OR "shock lung":ti,ab

#2 "acute respiratory distress":ti,ab OR "acute respiratory failure":ti,ab

#3 Acute:ti,ab AND ((respirat*:ti,ab OR ventilat*:ti,ab OR pulmon*:ti,ab) AND (fail*:ti,ab OR depression:ti,ab))

#4 [mh "lung injury"] OR ALI:ti,ab OR "Acute lung injury":ti,ab OR "Ventilator-Induced Lung Injury":ti,ab

#5 [mh "Respiratory insufficiency"] OR "Respiratory insufficiency":ti,ab

#6 [mh "Acute chest syndrome"] OR "Acute chest syndrome":ti,ab

#7 {OR #1-#6}

#8 [mh "Positive Pressure Respiration"] OR "Positive Pressure Respiration":ti,ab OR "Positive End-Expiratory Pressure":ti,ab OR PEEP:ti,ab

#9 "Continuous Positive Airway Pressure":ti,ab OR CPAP:ti,ab OR NCPAP:ti,ab

#10 "Airway Pressure Release Ventilation":ti,ab OR APRV:ti,ab

#11 "Intermittent Positive-Pressure Breathing":ti,ab OR IPPB:ti,ab

#12 "Intermittent Positive-Pressure Ventilation":ti,ab OR IPPV:ti,ab

#13 "lung protective ventilatory strategy":ti,ab OR LPVS:ti,ab

#14 "Alveolar recruitment":ti,ab OR "recruitment maneuvers":ti,ab

#15 {OR #8-#14}

#16 #7 AND #15

#17 [mh animals] not [mh humans]

#18 #16 not #17

Embase search strategy (until June 28, 2020)

S1 (EMB.EXACT("adult respiratory distress syndrome")) OR (TI,AB(ARDS OR "shock lung"))

S2 (TI,AB("acute respiratory" p/0 (distress OR failure*)))

S3 (TI,AB(acute n/3 (respirat* OR ventilat* OR pulmon*) n/3 (fail* OR depression)))

S4 (EMB.EXACT("acute lung injury")) OR (EMB.EXACT("hyperoxia-induced lung injury") OR EMB.EXACT("lung injury")) OR (EMB.EXACT("ventilator induced lung injury")) OR (TI,AB(ALI OR ("acute lung" p/0 injur*) OR "ventilator-Induced lung injury"))

S5 (EMB.EXACT.EXPLODE("respiratory failure")) OR (TI,AB(respiratory p/0 insufficien*))

S6 (EMB.EXACT("acute chest syndrome")) OR (TI,AB("acute chest syndrome"))

S7 (S1 OR S2 OR S3 OR S4 OR S5 OR S6)

S8 ((EMB.EXACT("positive end expiratory pressure")) OR (TI,AB("positive pressure respiration" OR "positive end-expiratory pressure" OR PEEP)))

S9 (TI,AB("continuous positive airway pressure" OR CPAP OR NCPAP))

S10 (TI,AB("airway pressure release ventilation" OR APRV))

S11 (TI,AB("intermittent positive-pressure breathing" OR IPPB))

S12 (TI,AB("intermittent positive-pressure ventilation" OR IPPV))

S13 (TI,AB(("lung protective" p/0 ventilat* p/0 strateg*) OR LPVS))

S14 (TI,AB((alveolar p/0 recruit*) OR (recruitment p/0 maneuve*)))

S15 (S8 OR S9 OR S10 OR S11 OR S12 OR S13 OR S14)

S16 (S7 AND S15)

S17 (((EMB.EXACT("controlled clinical trial") OR EMB.EXACT.EXPLODE("clinical trial (topic)") OR EMB.EXACT("randomized controlled trial")) OR (TI,AB(randomized) OR TI,AB(randomly) OR TI(trial))) NOT (ANIMAL(YES) NOT HUMAN(YES)))

S18 (S16 AND S17)

CINAHL search strategy (until July 12, 2020)

#1 (MH "Respiratory Distress Syndrome, Adult") OR TI ARDS OR AB ARDS OR TI "shock lung" OR AB "shock lung"

#2 TI "acute respiratory distress" OR AB "acute respiratory distress" OR TI "acute respiratory failure" OR AB "acute respiratory failure"

#3 TI Acute OR AB Acute AND ((TI respirat* OR AB respirat* OR TI ventilat* OR AB ventilat* OR TI pulmon* OR AB pulmon*) AND (TI fail* OR AB fail* OR TI depression OR AB depression))

#4 (MH "Lung injury+") OR TI ALI OR AB ALI OR TI "Acute lung injur*" OR AB "Acute lung injur*" OR TI "Ventilator-Induced Lung Injury" OR AB "Ventilator-Induced Lung Injury"

#5 (MH "Respiratory Failure+") OR TI "Respiratory Failure" OR AB "Respiratory Failure"

#6 (MH "Acute chest syndrome") OR TI "Acute chest syndrome" OR AB "Acute chest syndrome"

#7 S1OR S2 OR S3 OR S4 OR S5 OR S6

#8 (MH "Positive-Pressure Respiration+") OR TI "Positive Pressure Respiration" OR AB "Positive Pressure Respiration" OR TI "Positive End-Expiratory Pressure" OR AB "Positive End-Expiratory Pressure" OR TI PEEP OR AB PEEP

#9 TI "Continuous Positive Airway Pressure" OR AB "Continuous Positive Airway Pressure" OR TI CPAP OR AB CPAP OR TI NCPAP OR AB NCPAP

#10 TI "Airway Pressure Release Ventilation" OR AB "Airway Pressure Release Ventilation" OR TI APRV OR AB APRV

#11 TI "Intermittent Positive-Pressure Breathing" OR AB "Intermittent Positive-Pressure Breathing" OR TI IPPB OR AB IPPB

#12 TI "Intermittent Positive-Pressure Ventilation" OR AB "Intermittent Positive-Pressure Ventilation" OR TI IPPV OR AB IPPV

#13 TI "lung protective ventilatory strateg*" OR AB "lung protective ventilatory strateg*" OR TI LPVS OR AB LPVS

#14 TI "alveolar recruit*" OR AB "alveolar recruit*" OR TI "recruitment maneuve*" OR AB "recruitment maneuve*"

#15 S8 OR S9 OR S10 OR S11 OR S12 OR 13 OR S14

#16 S7 AND S15

#17 (MH "Randomized Controlled Trials")

#18 TI ( random* or placebo* or blind* or double blind* ) OR AB ( random* or placebo* or blind* or double blind* )

#19 S17 OR S18

#20 S16 AND S19
